# Pegargiminase Suppresses the Fanconi Anemia Pathway and Promotes Melphalan‐Induced DNA Double‐Strand Breaks in Uveal Melanoma

**DOI:** 10.1111/pcmr.70104

**Published:** 2026-06-19

**Authors:** Iuliia Pavlyk, George Field, Matthew Young, Josephine Carpentier, Emilia A. Szlosarek, Michaela R. O'Keeffe‐Brown, Timothy Crook, Nelofer Syed, John S. Bomalaski, Pui Ying Chan, Peter W. Szlosarek

**Affiliations:** ^1^ Center for Cancer Biomarkers and Biotherapeutics, Barts Cancer Institute (BCI)—A Cancer Research UK Center of Excellence, John Vane Science Center Queen Mary University of London London UK; ^2^ University of East Anglia Norwich UK; ^3^ The London Clinic London UK; ^4^ Department of Brain Sciences, Hammersmith Hospital Imperial College London London UK; ^5^ Polaris Pharmaceuticals Inc. San Diego California USA; ^6^ Wellcome Sanger Institute Hinxton UK; ^7^ Department of Medical Oncology, Barts Health NHS Trust St Bartholomew's Hospital London UK

**Keywords:** ADI‐PEG20, arginine dependency, DSBs, Fanconi anemia, melphalan, pegargiminase, uveal melanoma

## Abstract

Uveal melanoma is a hard‐to‐treat arginine‐dependent cancer secondary to argininosuccinate synthetase 1 (ASS1) loss with half of patients succumbing to liver‐dominant metastases. Arginine deprivation with pegargiminase is a novel antimetabolite strategy for patients with uveal melanoma. We investigated the preclinical rationale for combining pegargiminase with melphalan, an alkylating agent approved recently for the treatment of hepatic‐centric disease. Drug sensitivity of ASS1‐deficient uveal melanoma cell lines was performed in 2D culture using proliferation and cytotoxicity assays, with analysis of cell death, cell cycle, DNA double‐strand breaks, and interrogation of the molecular mechanism of action by RNA‐seq. ADI‐PEG20 and melphalan suppressed uveal melanoma cell line proliferation and triggered cytotoxicity, effects which were enhanced with the drug combination. ADI‐PEG20 downregulated multiple genes of the Fanconi anemia pathway and synergized with melphalan to increase DNA double‐strand breaks. Melphalan and pegargiminase is a rational new drug combination that warrants clinical testing in uveal melanoma.

## Significance

- Uveal melanoma is an arginine‐dependent cancer secondary to ASS1 loss and is sensitive to arginine deprivation with pegargiminase (ADI‐PEG20).
- Here, we show that pegargiminase suppresses multiple Fanconi anemia pathway genes and synergizes to increase DNA double‐strand breaks in uveal melanoma cells, with enhanced cytotoxicity and PARP and caspase‐dependent apoptosis.
- Clinical trials of pegargiminase plus melphalan are warranted in uveal melanoma.

## Graphical abstract

ADI‐PEG20 enhances melphalan‐induced DNA DSBs in UM cells. We describe potentiation of DNA DSBs in UM cell lines upon treatment with ADI‐PEG20 and melphalan (92.1 cells with STRIDE by intoDNA). ADI‐PEG20 suppresses multiple members of the FA pathway—a known resistance mechanism for melphalan—providing a rationale for exploring this drug combination in the clinic.

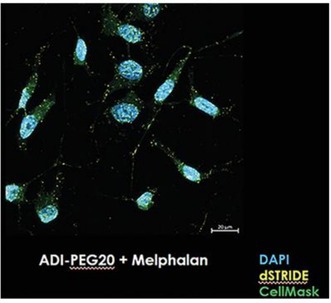

## 1 Introduction

Uveal melanoma (UM) is the commonest ocular malignancy in adulthood known for poor survival outcomes secondary to multifocal liver metastases and liver failure developing in approximately 50% of patients (Jager et al. 2020). Treatments are limited and include immune checkpoint blockade (ICB) and tebentafusp, with clinical benefit observed in a subset of patients (Hassel et al. 2023; Pelster et al. 2021). The median overall survival is 10–22 months, with 5‐year survivals remaining low at around 10%–20% compared with over 50% in cutaneous metastatic melanoma with immunotherapy over the last decade (Dimitriou et al. 2025; Khoja et al. 2019; Lane et al. 2018; Wolchok et al. 2022).

Local therapies, including liver metastasectomy and radio‐frequency ablation provide benefits to selected patients with oligometastatic disease (Hand et al. 2020). More recently, chemosaturation with percutaneous hepatic perfusion of melphalan has been approved by the FDA for metastatic UM, which is also being studied in the context of immunotherapy (Olofsson Bagge et al. 2024). Melphalan, as a classic alkylating agent, induces DNA damage, thereby potentially enhancing antigenicity for ICB therapy (Patel and Minn 2018).

Previously, we and others reported an encouraging 60%–70% disease control rate in patients with UM using the arginine‐depleting enzyme, pegylated arginine deiminase (ADI‐PEG20, pegargiminase) (Chan et al. 2022; Ott et al. 2013). ADI‐PEG20 exploits the known arginine auxotrophy of argininosuccinate synthetase 1 (ASS1) cancers of which UM is an exemplar (Delage et al. 2010). We have shown that ADI‐PEG20 potentiates the effect of antifolate chemotherapy and increases overall survival in patients with ASS1‐deficient mesothelioma, but not in UM (Allen et al. 2014; Chan et al. 2022; Szlosarek et al. 2024). Thus, alternative combinations of ADI‐PEG20 in UM are needed. Here, we explored the preclinical rationale for combining ADI‐PEG20 with melphalan using a human UM cell line panel. Our data highlight a novel mechanism of action involving double‐strand breaks (DSBs) in DNA and support further clinical development of ADI‐PEG20 in the context of DNA‐damaging agents.

## 2 Results

### 2.1 Increased Antiproliferative Activity of ADI‐PEG20 Plus Melphalan in UM Cell Lines

**FIGURE 1:**
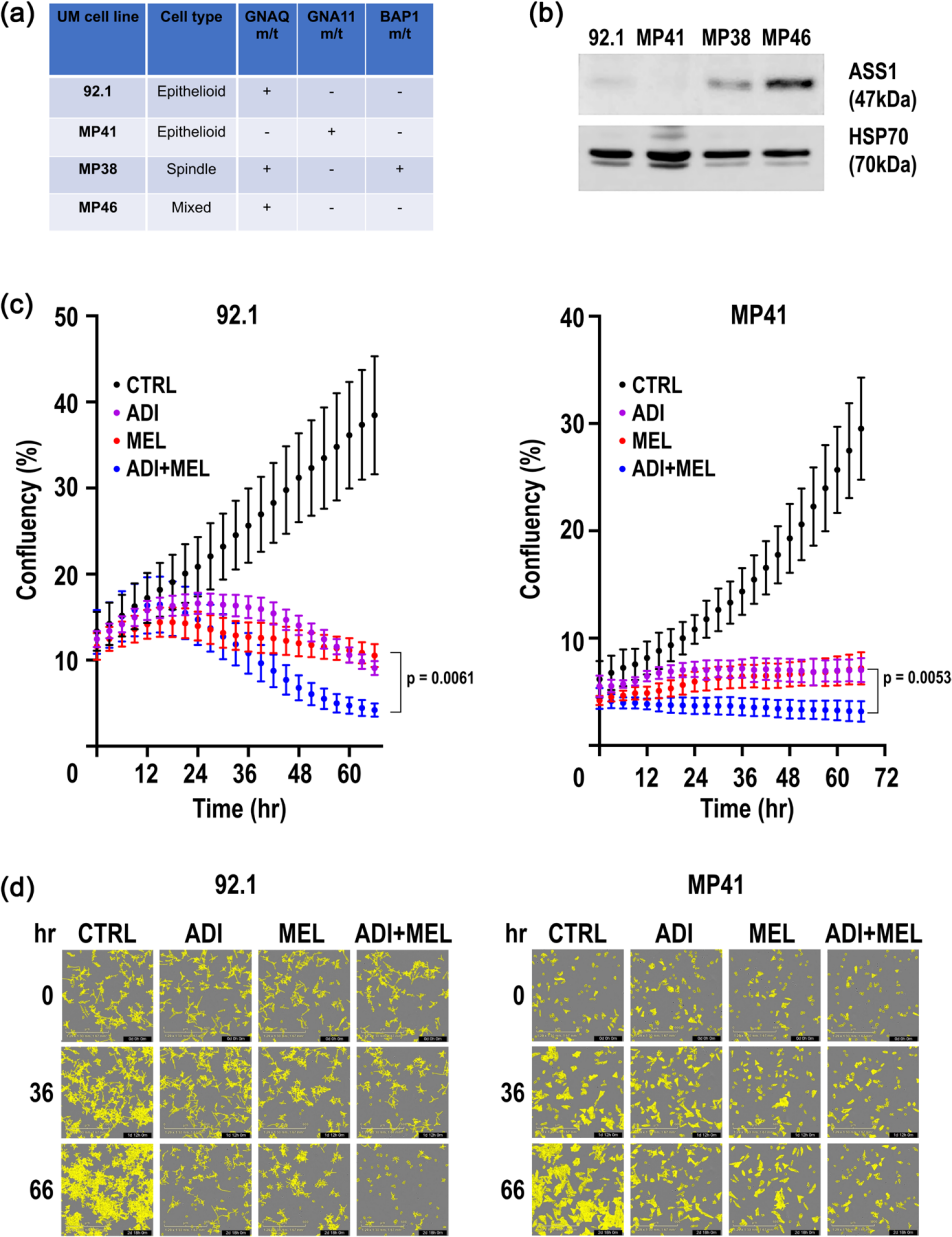
ADI‐PEG20 and melphalan inhibit proliferation of ASS1‐deficient UM cell lines. (a) Mutation status. (b) ASS1 western blotting. (c) Cell proliferation in 92.1 cell line and MP41 cell lines with melphalan (0.77 μM) and ADI‐PEG20 (500 ng/mL) alone or in combination and compared to untreated cells (representative of three experiments performed in triplicate). (d) Typical cell confluency images of 92.1 and MP41 respectively at *t* = 0, 36, and 66 h for assessment of proliferation. Scale bar 600 μm.

First, we screened a panel of four UM cell lines identifying ASS1 deficiency in 92.1 and MP41, while MP38 and MP46 were ASS1‐proficient (Figure 1a,b). We tested the proliferation of the ASS1‐deficient cell lines in 2D culture by analyzing cell confluency using an IncuCyte imaging platform. As expected, ADI‐PEG20 significantly inhibited the growth of the 92.1 and MP41, while melphalan also displayed growth‐suppressive activity. Notably, the combination of ADI‐PEG20 and melphalan was at least additive in both the 92.1 and MP41 (Figure 1c,d) cell lines.

### 2.2 Increased Cytotoxicity and Apoptosis of 92.1 UM Cells With ADI‐PEG20 and Melphalan

**FIGURE 2:**
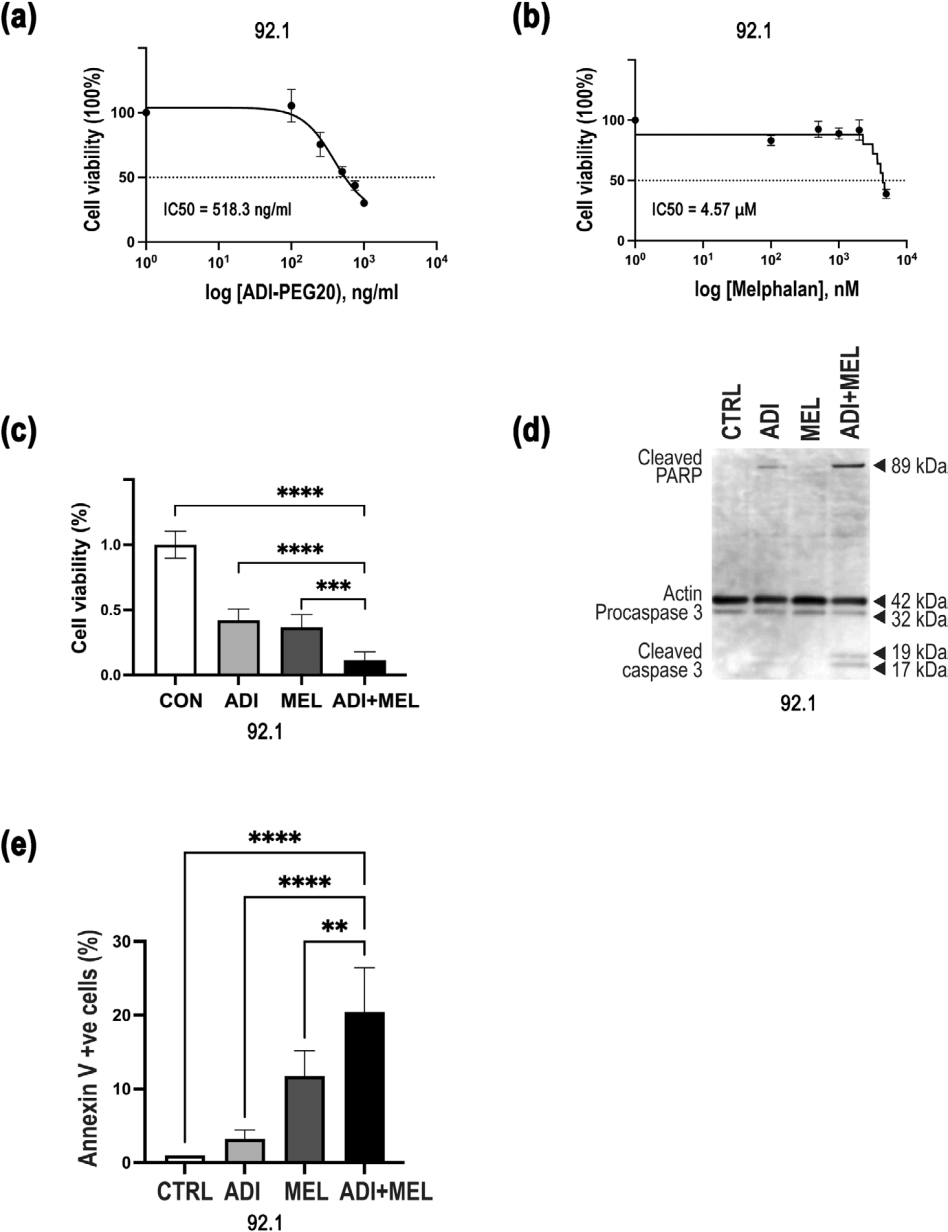
ADI‐PEG20 and melphalan increase 92.1 cell line cytotoxicity and apoptosis. (a) IC50 for ADI‐PEG20 (48 h; *n* = 3). (b) IC50 for melphalan (48 h; *n* = 3). (c) Cell viability for control, ADI‐PEG20 (500 ng/mL), melphalan (5 μM), and the drug combination (*n* = 9) with **p < 0.001, ***p = 0.003, and ****p < 0.0001. (d) Immunoblotting for PARP and caspase 3 following treatment with control, ADI‐PEG20 (500 ng/mL), melphalan (5 μM), and the drug combination. (e) Analysis of Annexin V positive cells by flow cytometry upon treatment with control, ADI‐PEG20 (500 ng/mL), melphalan (5 μM), and the drug combination (*n* = 6) with **p < 0.001 and ****p < 0.0001.

Next, we determined the IC50 of ADI‐PEG20 and melphalan in the ASS1‐deficient UM cell lines (Figure 2a,b) and analyzed cell viability confirming increased cytotoxicity of the combination by 48 h in the 92.1 cell line but not the MP41 cell line (Figure 2c; Figure S1). Cell death was caspase‐dependent and poly(ADP‐Ribose) polymerase (PARP)‐dependent in the 92.1 cell line (Figure 2d) and was validated by Annexin V staining as a measure of increased apoptosis in the ADI‐PEG20 and melphalan combination compared to either drug alone (Figure 2e). There was no increased cleaved PARP or caspase‐3 with ADI‐PEG20 plus melphalan in MP41 cells indicating differential drug effects despite common ASS1 deficiency in the two cell lines (Figure S2).

### 2.3 ADI‐PEG20 Promotes DSBs by Melphalan With S and G2/M Cell Cycle Arrest in 92.1 Cells

**FIGURE 3:**
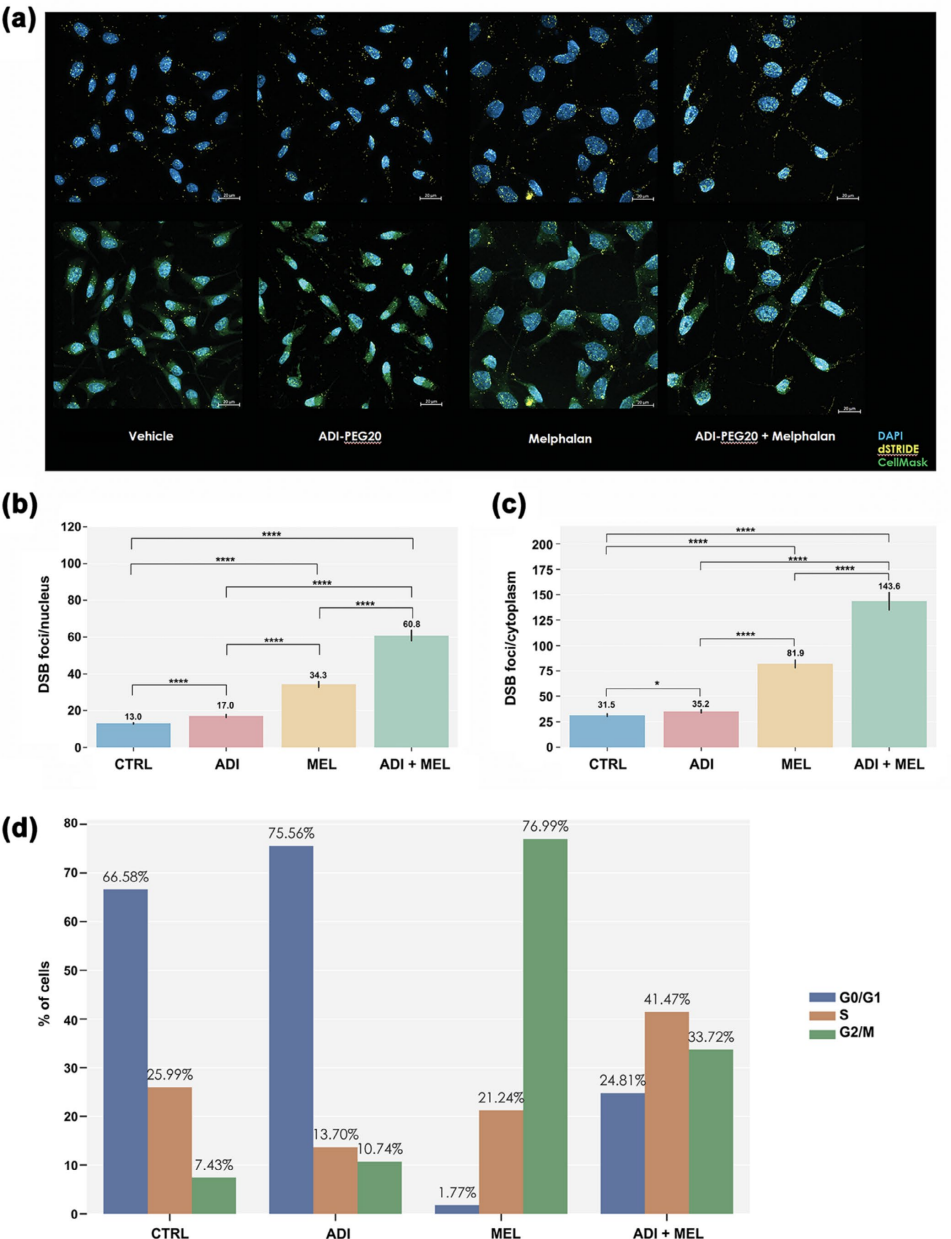
ADI‐PEG20 enhances melphalan‐induced DNA DSBs in 92.1 cells. (a) DNA DSBs revealed as yellow fluorescent foci by confocal microscopy (scale bar 20 μm). (b) DSBs in the nucleus. (c) DSBs in the cytoplasm. (d) Percentage of cells in a given cell cycle phase. *p < 0.05 and ****p < 0.0001.

Since melphalan is a well‐known DNA‐damaging alkylating agent, we addressed the further impact of pegargiminase by analyzing the extent of DSBs in DNA. Our analysis revealed that indeed melphalan induced a significant increase in DSBs, whereas this was minimal with pegargiminase, but that there was a fivefold synergistic increase in DSBs compared to control in UM cells treated with the combination of ADI‐PEG20 and melphalan (Figure 3a–c). Moreover, there was a corresponding increase of DSBs detected in the cytoplasm of pegargiminase and melphalan treated UM cells. Cell cycle analysis was based on nuclear surface and volume and DAPI intensity, and indicated cell cycle arrest at the G2/S phase with melphalan alone and the drug combination, whereas ADI‐PEG20 triggered arrest at G0/G1 (Figure 3d). Moreover, cells in G2/M and S phases were characterized by higher dSTRIDE counts when compared to G0/G1 phases (Figure S3). Similarly, there was a pronounced increase in the dSTRIDE counts in the cytoplasm of UM cells in G2/M and S phases treated with ADI‐PEG20 and melphalan compared with control, melphalan, or ADI‐PEG20 (Figure S4).

### 2.4 Major Downregulation of the Fanconi Anemia Pathway by ADI‐PEG20 in 92.1 Cells

**FIGURE 4:**
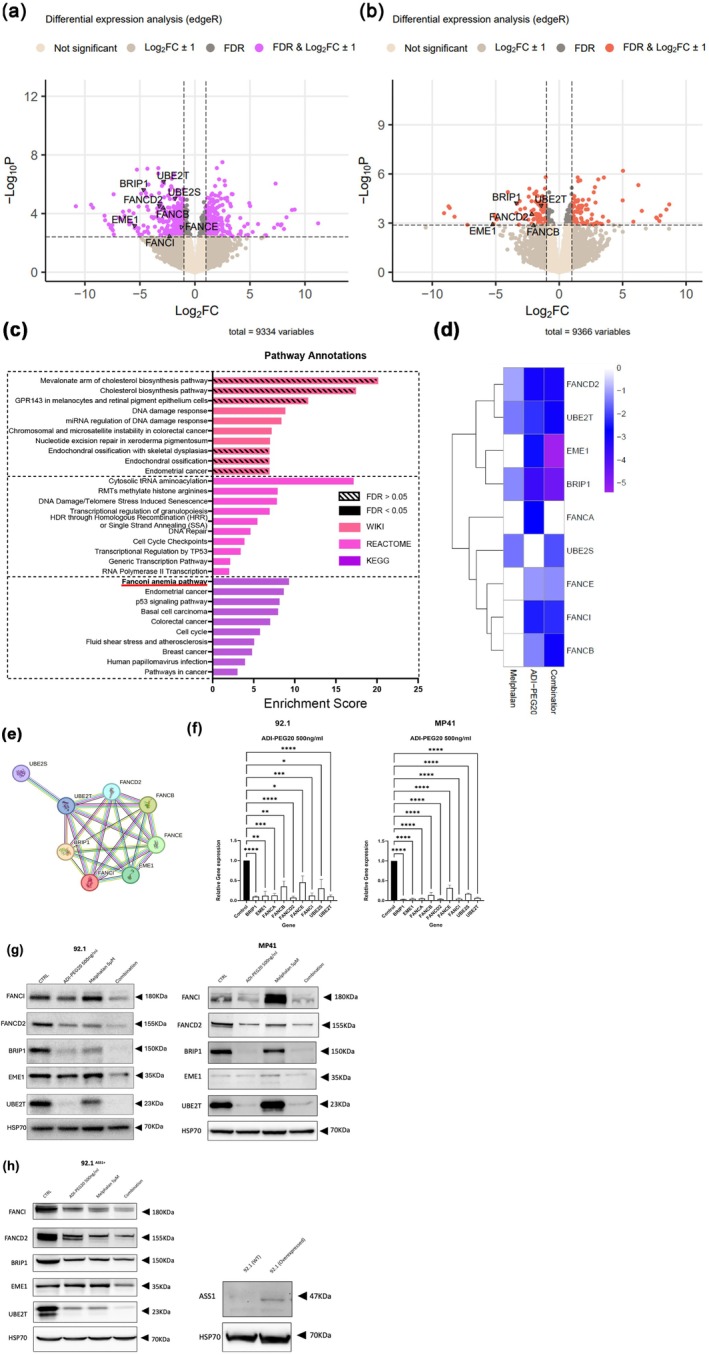
ADI‐PEG20 suppresses genes of the FA pathway in 92.1 cells. Volcano plots of the differentially expressed genes of 92.1 cell line treated with (a) the combination of ADI‐PEG20 and melphalan compared to control, and (b) the combination compared to melphalan only; vertical lines illustrate differentially expressed genes with a log_2_ fold change of ±1; and horizontal lines describe the genes that where significantly differentially expressed at a false discovery rate of *q* < 0.05 by Benjamini–Hochberg method. (c) Pathway overrepresentation analyses of the differentially expressed genes identified in the comparison of combination treatment against melphalan treatment (volcano plot b); WebGestalt used an FDR of *q* < 0.05 within the Benjamini–Hochberg method to determine significantly overrepresented pathways in the KEGG, REACTOME, and WIKI databases. (d) Heatmap highlighting the differential expression of genes associated with the KEGG pathway annotation FA pathway in each of the treatment groups relative to untreated. (e) String database protein–protein interactions of the FA pathway hits from the combination against untreated differential expression analysis in (a). (f) qPCR analysis of FA pathway genes in 92.1 and MP41 cells treated with ADI‐PEG20 (500 ng/mL over 48 h). Relative expression levels normalized to housekeeping gene, PPIA. Bars represent the mean ± SEM (*n* = 3); *p < 0.05, **p < 0.01, ***p < 0.001, ****p < 0.0001, one‐way ANOVA with Dunnett's Multiple comparison test. (g) Representative western blot analysis of 92.1 and MP41 cells showing protein levels of FANCI, FANCD2, BRIP1, EME1, and UBE2T treated with ADI‐PEG20 (500 ng/mL), Melphalan (5 μM), and the combination (*n* = 3); HSP70 is shown as a loading control. (h) Representative western blot analysis of ASS1‐overexpressing 92.1 cells showing protein levels of FANCI, FANCD2, BRIP1, EME1, and UBE2T treated with ADI‐PEG20 (500 ng/mL), Melphalan (5 μM), and the combination (*n* = 3); HSP70 is shown as a loading control. ASS1 protein expression after lentiviral transduction in 92.1 is shown on the right‐hand side; HSP70 is shown as a loading control.

To understand the mechanism underlying the increased DSBs with ADI‐PEG20 and melphalan, we employed RNA sequencing (RNA‐seq). Bioinformatic analysis by WIKI revealed modulation of the DNA damage response pathway and by Reactome modulation of DNA repair, transcriptional regulation by p53 and cell cycle checkpoint pathways. Several KEGG database pathways were also altered in the drug combination including p53 signaling and the cell cycle; however, the most represented and statistically significant was widespread suppression of the Fanconi Anemia (FA) pathway (Figure 4a). Furthermore, ADI‐PEG20 and not melphalan was the key drug downregulating the FA genes: *FANCD2*, *UBE2T*, *EME1*, *BRIP*, *FANCA*, *FANCE*, *FANCI*, and *FANCB* (Figure 4b–d). A protein–protein interaction of the FA pathway was derived in response to the drug combination compared to the control treatment (Figure 4e).

We validated the FA pathway as the top pathway regulated by ADI‐PEG20 in the 92.1 cell line by qPCR (Figure 4f) and western blotting (Figure 4g,h). Notably, we identified that ADI‐PEG20 was critical in reducing the expression of multiple FA proteins—with a modest inhibitory effect of melphalan on FANCD2 and BRIP1 levels—and that the combination of ADI‐PEG20 and melphalan had a further suppressive and cytotoxic effect in 92.1 but not in MP41 cells. Additionally, ASS1 overexpression in 92.1 cells abrogated the ADI‐PEG20‐mediated suppression of the FA pathway, confirming that the downregulation of the FA pathway in response to arginine deprivation is ASS1‐dependent.

## 3 Discussion

Chemosaturation with melphalan is an FDA approved therapy for hepatic‐dominant UM, a disease that remains an area of high unmet need with a minority of patients surviving 5 years. In this study, we have shown that arginine deprivation with pegargiminase downregulates multiple components of the FA pathway and potentiates the DNA‐damaging effects of melphalan as evidenced by a marked increase in DNA DSBs. UM cells characterized by ASS1 loss were highly sensitive to the combination of ADI‐PEG20 and melphalan triggering arrest in G2/M and S phase and apoptosis.

Previous studies in hematological cancers have shown that the FA pathway is a key driver of melphalan resistance (Yarde et al. 2009). In solid cancers, such as pancreatic cancer, deficiency of several FANC genes increased tumor cell susceptibility specifically to interstrand crosslinkers, including melphalan, cisplatin, and mitomycin‐C (van der Heijden et al. 2005). In contrast, cancer cells with FA‐deficient genes were insensitive to a range of non‐alkylating chemotherapeutic agents, including gemcitabine, 5‐fluorouracil, vinorelbine, and paclitaxel. The *FANCI* gene downregulated by ADI‐PEG20 in our study is critical for DNA repair via *FANCD2* heterodimerization, and their inhibition leads to DNA damage and chemosensitization (Li et al. 2022; Smogorzewska et al. 2007).

More broadly, the FA genes uncovered using RNA‐Seq are part of the homologous recombination (HR) repair pathway, with mutations directly impacting HR repair mechanisms that lead to genomic instability, cancer predisposition and increased chemotherapy‐associated toxicities (Niraj et al. 2019). Additionally, germline and somatic FA gene mutations in cancer have been linked to improved chemotherapy outcomes in patients with several solid cancers exposed to alkylating agents, including breast, pancreatic, bladder, and liver cancer (Lai et al. 2022; O'Reilly et al. 2020; Plimack et al. 2024; Rodler et al. 2023). A recent study of germline variants in patients with melanoma reported on uncommon alterations in *BRIP1* (1/83) and *FANCE* (1/83) with a pathogenic role in UM tumorigenesis (Johansson et al. 2025). Defects in the FA pathway genes may explain sporadic responses reported to alkylating drugs in UM akin to responses described for gemcitabine in *MBD4*‐mutated UM (Chabot et al. 2022; Schinzari et al. 2017).

Studies of arginine deprivation over the last two decades have revealed a cohort of cancers with substantial ASS1 deficiency and sensitivity to the arginine‐catabolizing enzymes ADI‐PEG20 and arginase (Field et al. 2023). Previously, we showed that UM displays near universal loss of ASS1 with evidence of promoter methylation in metastatic disease (Chan et al. 2022). Here, the ASS1‐negative MP41 and 92.1 cell lines displayed reduced cell proliferation and viability to ADI‐PEG20; however, only the latter exhibited significant apoptosis upon drug combination therapy that was caspase and PARP‐dependent. Cell type is a key determinant of ADI‐PEG20 sensitivity, with variable effects noted across and between cell line studies (Lin et al. 2017; Zheng et al. 2013). Moreover, several ADI‐PEG20 resistance mechanisms have been identified, ranging from upregulation of ASS1—reported in patients with UM progressing on pegargiminase—and the antiapoptotic protein Bcl‐xL to activation of autophagy and the supply of the salvage precursor argininosuccinate via tumor‐associated macrophages (Chan et al. 2022; Kim et al. 2009; Panda et al. 2025; Phillips et al. 2023).

ADI‐PEG20 plus melphalan adds to the number of rational combinations that exploit synergies as well as nonredundant anticancer drug mechanisms incorporating arginine deprivation. To date, we have succeeded in translating pegargiminase with pemetrexed plus platinum for the treatment of chemorefractory mesothelioma. This exploits the inhibitory effect of ADI‐PEG20 on key enzymes involved in thymidine synthesis thereby enhancing pemetrexed cytotoxicity in mesothelioma—the first arginine‐lowering chemotherapy to seek FDA approval (Szlosarek et al. 2024). A global phase 3 study is underway of another antimetabolite‐based regimen, namely ADI‐PEG20 with gemcitabine and docetaxel in the therapy of ASS1‐deficient soft tissue sarcoma. This chemotherapy regimen capitalizes on the taxane and ADI‐PEG20 stabilizing hENT1 surface expression resulting in enhanced gemcitabine uptake and tumor cytotoxicity (Prudner et al. 2019). The alkylating agent temozolomide also enhances the effects of ADI‐PEG20 with the combination now in frontline clinical trials for patients receiving surgery and radiation for glioblastoma multiforme (Przystal et al. 2018). Therefore, the application of chemosaturation therapy with melphalan, which delivers a 10–100‐fold higher peak perfusate drug concentration than systemic dosing and has the single highest drug activity in uveal melanoma of up to 60%, merits further study in the context of arginine depletion using pegargiminase and also immunotherapies (Artzner et al. 2019; Chang et al. 2021; Kraehenbuehl et al. 2022; Minor et al. 1985).

Since our drug combination data were confined to only one of two ASS1‐deficient UM cell lines, namely 92.1, we expanded our ASS1 negative UM cell line panel to include Mel270 and confirmed decreased cell proliferation and viability, and increased DNA DSBs with ADI‐PEG20 and melphalan co‐treatment compared with monotherapy (Figure S5). However, unlike 92.1 cells which demonstrated caspase 3 and PARP cleavage with the drug combination, this was not observed in Mel270 cells indicating likely caspase‐independent cell death. The latter mechanism of cell death has been reported with arginine‐degrading enzymes in various solid cancer cell lines (Field et al. 2023). Further functional validation of our data with *ASS1* knockdown/knockout in the MP38 and MP46 cell lines was not possible due to extremely poor growth in vitro consistent with the known tumor suppressive role of ASS1. Additionally, further study of the ADI‐PEG20‐depleted FA pathway is needed to address which gene combination is critical to the increased melphalan sensitivity. Lastly, although there was no enhanced cytotoxicity nor apoptosis from adding ADI‐PEG20 to melphalan in the MP41 UM cell line, alternative druggable pathways with arginine deprivation deserve scrutiny. In particular, the known activating mutations of GNAQ or GNA11 sensitize UM to the PKC inhibitor darovasertib currently in clinical trials (Visser et al. 2024). Bypass mechanisms of resistance for PKC in UM include PI3K which is also a driver of ASS1 induction in melanoma via c‐Myc, thus providing a rationale for studying ADI‐PEG20 with kinase inhibition, which is an expanding area in UM therapeutics (Park et al. 2024; Tsai et al. 2012; Baqai et al. 2023).

Collectively, our data provide a novel and readily translatable drug combination therapy for liver‐dominant metastases from uveal melanoma that exploits inhibition of DNA repair by ADI‐PEG20 thereby potentiating melphalan cytotoxicity. A clinical trial of pegargiminase plus hepatic chemosaturation with melphalan is planned (ATOMIC‐UM).

## 4 Materials and Methods

### 4.1 Cell Culture and Drug Treatments

UM cell lines were purchased from ATCC and maintained in endotoxin‐free RPMI 1640 medium (Gibco, #61870010) for 92.1 and MP4, and DMEM (Gibco, #10566016) for MP46 and MP38, supplemented with 10% (v/v) heat‐inactivated fetal bovine serum (Gibco, #16140071) and 1% (v/v) penicillin–streptomycin. Cells were cultured at 37°C in a humidified incubator with 5% CO_2_. All cell lines were checked regularly for Mycoplasma contamination. ADI‐PEG20 was from Polaris Pharmaceuticals Inc. (San Diego, USA). Melphalan was purchased from Cambridge Bioscience (#16665). All drug treatments were performed 24 h following cell plating, when medium was replaced with fresh medium alone or with ADI‐PEG20 (500 ng/mL), melphalan (5 μM unless specified otherwise), or the drug combination for 48 h. Relevant controls and validation of ADI‐PEG20 have been performed previously within our group (Phillips et al. 2023; Szlosarek et al. 2024).

### 4.2 2D IncuCyte Live Cell Proliferation Assay

Cells were trypsinized and seeded at 2 × 10^3^ cells/well in a 96‐well plate and grown overnight. Twenty‐four hours later, wells were replaced with fresh medium alone or with ADI‐PEG20 (500 ng/mL), melphalan (0.77 μM), or the drug combination. The plate was then placed into the IncuCyte S3TM live cell imaging system (Essen Bioscience, MI, USA), and images of well confluency were recorded every 2 h, up to a duration of 96 h. Well confluency was calculated automatically using the IncuCyte S3TM and then normalized against *t* = 0 h to obtain the proliferation rate.

### 4.3 Cell Viability Assay (CTG)

Cells were trypsinized and seeded at 2 × 10^3^ cells/well in a 96‐well plate and grown overnight prior to drug treatment. Cell viability was measured using the CellTiter‐Glo Cell Viability Assay (Promega, #G7571) at 48 h, according to the manufacturer's instructions. Percent cell viability was normalized to control untreated cells.

### 4.4 ASS1 Overexpression

ASS1 overexpressing 92.1 cells were generated using lentiviral transduction. Briefly, lentiviral particles were produced by transient co‐transfection of HeLa cells with ASS1 transcript variant 1 cloned into the pLenti‐P2A‐Puro Lentiviral Gene Expression Vector driven by the EF1α promoter (OriGene, #PS100142), together with the Lenti‐vpak packaging system (OriGene, #TR30037). Transfection was performed using TurboFectin 8.0 (OriGene) in a 6‐well plate format according to the manufacturer's protocol. Viral supernatants were harvested 48 h post‐transfection and passed through a 0.45 μm syringe filter to eliminate cellular debris. For lentiviral transduction, 92.1 cells were seeded in 10 cm dishes and incubated with the filtered lentiviral supernatant supplemented with 8 μg/mL polybrene (Sigma, #TR‐1003) for 18 h. Following transduction, the medium was replaced with fresh complete medium, and cells were subsequently expanded in 10 cm dishes and subjected to treatment with ADI‐PEG20, melphalan, or their combination as indicated. ASS1 overexpression was verified by western blot analysis using an anti‐ASS1 antibody (Cell Signaling Technology, #70720; dilution 1:1000).

### 4.5 Reverse Transcription Quantitative PCR (qPCR)

3 × 10^5^ cells were plated in 6‐well plates in 2 mL RPMI (10% FBS, 1% penicillin–streptomycin). Cells were treated with ADI‐PEG20 (500 ng/mL) or 1× PBS for 48 h. The Qiagen RNAeasy mini kit was used to extract RNA from 6‐well plates according to manufacturer protocol. After the RNA extraction, total RNA concentration and purity were determined using Nanodrop (ThermoFisher). Purity was determined by measuring the absorbance at 230, 260, and 280 nm. Only RNA samples with a 260/230 ratio and a 260/280 ratio above 1.5 were used. To generate cDNA, the High‐Capacity cDNA Reverse Transcription Kit (ThermoFisher, #4368814) was used as per manufacturer instructions. qPCR was performed using TaqMan probes using the QuantStudio Real‐Time PCR Systems (Applied Biosystems). All qPCR reactions were carried out in duplicate using MicroAmp Optical 96‐Well Reaction Plates (Applied Biosystems, Life Technologies) sealed with MicroAmp Optical Adhesive Films (Applied Biosystems, Life Technologies). Each well of the 96‐well reaction plate contained 0.5 μL of appropriate cDNA, 0.5 μL of appropriate TaqMan Probe, 4 μL of ddH_2_O, 5 μL TaqMan 2× Master Mix. List of RT‐qPCR probes are as follows: BRIP1 (ThermoFisher, #Hs00908143_m1), FANCA (ThermoFisher, #Hs01116668_m1), FANCB (ThermoFisher, #Hs00537483_m1), FANCD2 (ThermoFisher, #Hs00276992_m1), FANCE (ThermoFisher, #Hs01562763_m1), FANCI (ThermoFisher, #Hs00383049_m1), EME1 (ThermoFisher, #Hs01103357_g1), UBE2S (ThermoFisher, #Hs02797093_g1), UBE2T (ThermoFisher, #Hs00928040_m1), PPIA (ThermoFisher, #Hs04194521_s1). The ∆∆CT method was used for the analysis within GraphPad Prism.

### 4.6 Western Blot Analysis

Plates were seeded at a density of 2.2 × 10^6^ cells. Cells were lysed at 48 h following drug treatment in RIPA lysis buffer (50 mM Tris HCL [pH 8], 150 mM NaCl, 1% NP40, 1× protease inhibitor, deionized water) for 15 min. Lysates were centrifuged for 15 min at 12,000 rpm, and the supernatant was collected. Protein concentration was determined using the BSA protein assay (Bio Rad, #500‐0116). Samples were reduced with 10× reducing agent (ThermoFisher, #NP0009) and 4× LDS buffer (ThermoFisher, #NP0007). Protein samples were denatured on a heating block for 5 min at 95°C, separated by SDS‐PAGE, and transferred to a nitrocellulose membrane. Primary antibodies were used as follows: ASS1 (1:1000), apoptosis western blot cocktail (pro/p17‐caspase‐3, cleaved PARP1, muscle actin) (Abcam #136812), FANCD2 (1:3000, ThermoFisher, #MA1‐16570), FANCI (1:3000, ThermoFisher, #20789–1‐AP), EME1 (1:1000, ThermoFisher, #PA5‐79202), UBE2T (1:1000, ThermoFisher, #10105–2‐AP), BRIP1 (1:1000, ThermoFisher, #PA5‐27096) in 5% milk for 1 h at room temperature or overnight. After washing (3 times for 10 min in TBS‐T), the membranes were incubated in either anti‐rabbit or anti‐mouse Horseradish Peroxidase (HRP) conjugated secondary antibody (Dako anti‐rabbit #P0448 or anti‐mouse, Dako, #P0447) diluted at a concentration of 1:5000 in 5% milk for 1 h at room temperature. After incubation with the secondary antibody, the membranes were washed again three times for 10 min in TBS‐T. Immunoreactivity was detected using chemiluminescence substrate (SuperSignal West Pico Chemiluminescent substrate, Pierce, #32209) on Amersham Imager 600 Chemi‐doc. Expression levels of β‐Actin or HSP70 were used as protein loading controls.

### 4.7 Annexin Staining

Cells were trypsinized and seeded at 2.2 × 10^6^ cells/well in 10 cm plates and grown overnight prior to drug treatment. Forty‐eight hours later, cells were trypsinized, washed with PBS, and resuspended in 100 μL Annexin V binding buffer (Invitrogen, #V13241). Cells were stained with 5 μL of Alexa Fluor 488 Annexin V and 1 μL 100 μg/mL PI (Invitrogen, #V13241). Staining was analyzed by flow cytometry on a LSR Fortessa1 flow cytometer (BD Biosciences). Data were analyzed by FlowJo software (BD Biosciences).

### 4.8 dSTRIDE Analysis

Cells were seeded on cover glasses in 12‐well plates at a density 1 × 10^5^ cells/well and incubated overnight followed by drug treatment for 48 h. Next, all samples were fixed with ice‐cold 70% EtOH (Fisher Scientific, BP2818100) and stored at −20°C in preparation for dSTRIDE analysis by intoDNA (Krakow, Poland) (Kordon et al. 2020). The dSTRIDE procedure was performed followed by DAPI (ThermoFisher Scientific, #62248) staining. The coverslips with fixed cells were then mounted with Vectashield (Vector Laboratories, #H‐1000‐10) and stored at 4°C until imaging. Fields of view for imaging (*n* = 10 per sample) were chosen randomly over the coverslip surface. The images were then collected as three‐dimensional (3D) confocal stacks using Cell Discoverer 7 LSM 900 microscope, Zeiss.

### 4.9 RNA Sequencing (RNA‐Seq) Sample Processing

Cells were trypsinized and seeded at 0.3 × 10^6^ cells/well using 6‐well plates and grown overnight prior to drug treatment. Following 48 h of drug incubation, RNA was isolated using RNeasy Kit for RNA purification (Qiagen, #74104) and DNAase treated (Qiagen, #79254). Samples were sent to Imperial College for transcriptome library preparation and RNA‐seq.

### 4.10 Bioinformatics

Salmon alignment tool (v1.9.0) and GENCODE release 45 were used to quantify the transcript abundance estimates using the computing systems at Queen Mary University of London. Tximport was used to convert the abundance estimates into gene expression data with R in R‐Studio. edgeR (v4.0.16) was used for differential expression analysis. All significantly differentially expressed genes (FDR *q* < 0.05) that had a log_2_ fold change ≤ −1 or ≥ 1 were used of overrepresentation analyses using WEB‐based GEne SeT AnaLysis Toolkit (WEBGESTALT) for the Gene Ontology terms; Molecular Function, Biological Process, and Cellular Component. The pathway databases KEGG, PANTHER, and REACTOME were also investigated using this same method.

### 4.11 Statistical Analysis

All statistical analysis (mean ± SD) was undertaken in GraphPad Prism (version 10). Three independent experiments were performed unless indicated. Experiments with greater than two groups were analyzed by two‐way ANOVA unless specified otherwise. dSTRIDE data was analyzed using nonparametric Kruskal–Wallis and Dunn's Test. A *p* value of < 0.05 was considered statistically significant.

## Author Contributions

**Iuliia Pavlyk:** investigation, methodology, validation, writing – review and editing, formal analysis, supervision, data curation. **George Field:** investigation, writing – original draft, methodology, validation, writing – review and editing, software, formal analysis, data curation. **Matthew Young:** investigation, validation, writing – review and editing, formal analysis, data curation. **Josephine Carpentier:** investigation, methodology, validation, writing – review and editing, formal analysis, data curation, supervision. **Emilia A. Szlosarek:** investigation, validation, writing – review and editing. **Michaela R. O'Keeffe‐Brown:** investigation, validation, writing – review and editing. **Timothy Crook:** conceptualization, writing – review and editing. **Nelofer Syed:** resources, writing – review and editing. **John S. Bomalaski:** funding acquisition, writing – review and editing. **Pui Ying Chan:** resources, writing – review and editing. **Peter W. Szlosarek:** conceptualization, writing – original draft, writing – review and editing, validation, formal analysis, supervision, resources, data curation, project administration, visualization.

## Funding

This work was supported by a grant from Polaris Pharmaceuticals Inc. (MIMR1A3S).

## Conflicts of Interest

Peter W. Szlosarek is a recipient of research funding from Polaris Pharmaceuticals Inc., and John S. Bomalaski is an employee of Polaris Pharmaceuticals Inc. The other authors declare no conflicts of interest.

## Supporting information


**Figure S1:** Effect of ADI‐PEG20 and melphalan on MP41 cell line toxicity.
**Figure S2:** Analysis of PARP and caspase‐3 in MP41 cells.
**Figure S3:** Nuclear dSTRIDE foci count (mean values) according to 92.1 UM cell cycle phase.
**Figure S4:** Cytoplasmic dSTRIDE foci count (mean values) according to 92.1 UM cell cycle phase.
**Figure S5:** Additional validation in the ASS1 negative Mel270 cell line.

## Data Availability

The data that support the findings of this study are available on request from the corresponding author. The data are not publicly available due to privacy or ethical restrictions.
