# Supplementary material for: Pegargiminase Suppresses the Fanconi Anemia Pathway and Promotes Melphalan‐Induced DNA Double‐Strand Breaks in Uveal Melanoma
Source: Pigment Cell Melanoma Res. 2026 Jun 19;39(4):e70104. doi: 10.1111/pcmr.70104 (PMC13282448; doi:10.1111/pcmr.70104)
Supplement: Supplementary file 1 — Figure S1: Effect of ADI‐PEG20 and melphalan on MP41 cell line toxicity. Figure S2: Analysis of PARP and caspase‐3 in MP41 cells. Figure S3: Nuclear dSTRIDE foci count (mean values) according to 92.1 UM cell cycle phase. Figure S4: Cytoplasmic dSTRIDE foci count (mean values) according to 92.1 UM cell cycle phase. Figure S5: Additional validation in the ASS1 negative Mel270 cell line. [file PCMR-39-0-s001.docx]

**Fig. 1S: Effect of ADI-PEG20 and melphalan on MP41 cell line toxicity**

**
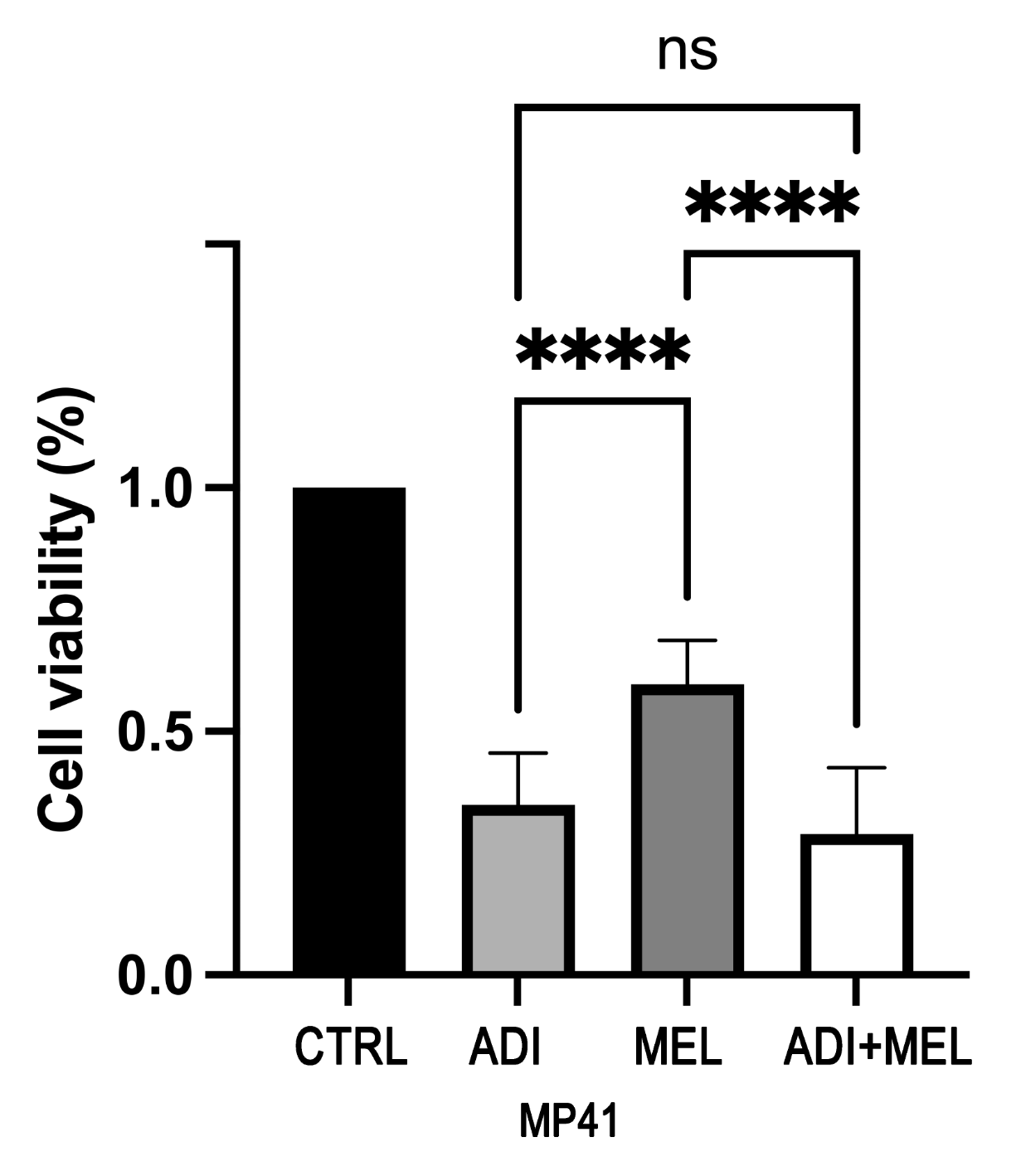
**

MP41 cells were treated with ADI-PEG20, melphalan, or the combination for 48hrs. Cell viability was assessed relative to untreated control (CTRL). Data are presented as mean ± SD from three biological replicates.

**Fig. 2S: Analysis of PARP and caspase-3 in MP41 cells**

**
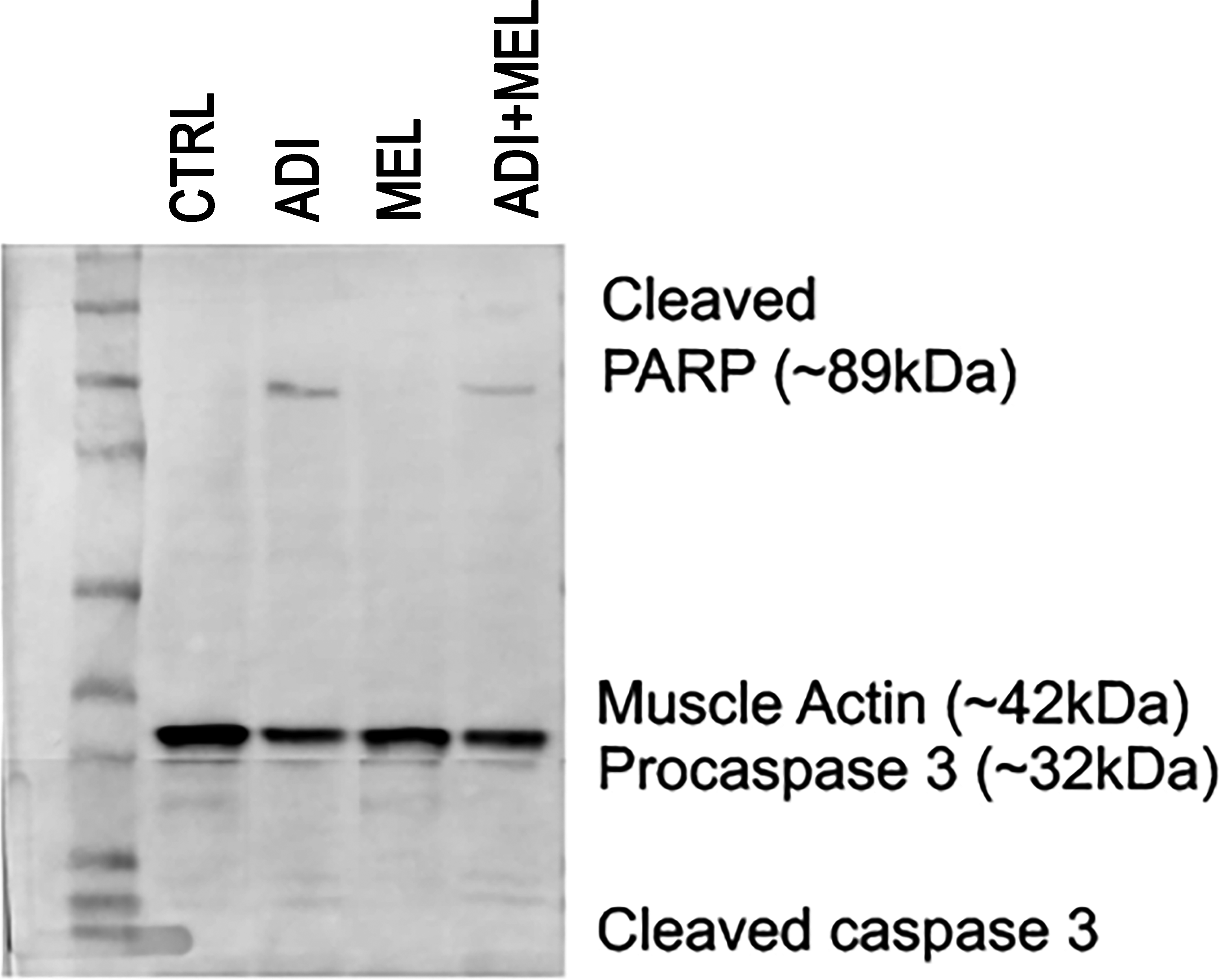
**

**Fig. 3S: Nuclear dSTRIDE foci count (mean values) according to 92.1 UM cell cycle phase**


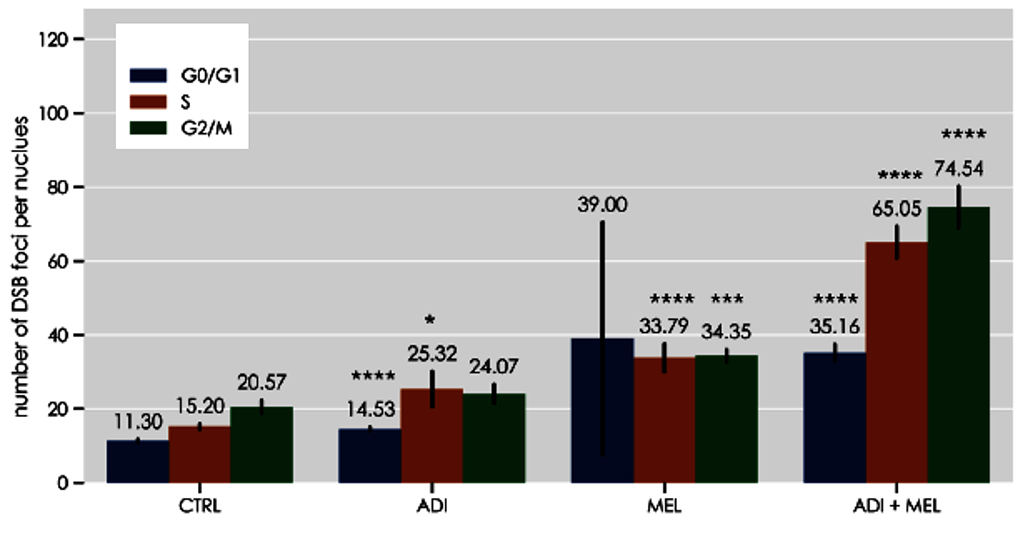


The number of nuclear dSTRIDE foci per nucleus was quantified in 92.1 uveal melanoma cells in G0/G1, S, and G2/M cell cycle phases after treatment with ADI-PEG20 (ADI), melphalan (MEL), or their combination. Untreated cells served as control (CTRL). Bars represent mean values for each condition and cell cycle phase, with error bars indicating variability among technical replicates. *p < 0.05; **p < 0.01; ***p < 0.001; ****p < 0.0001.

**Fig. 4S: Cytoplasmic dSTRIDE foci count (mean values) according to 92.1 UM cell cycle phase**


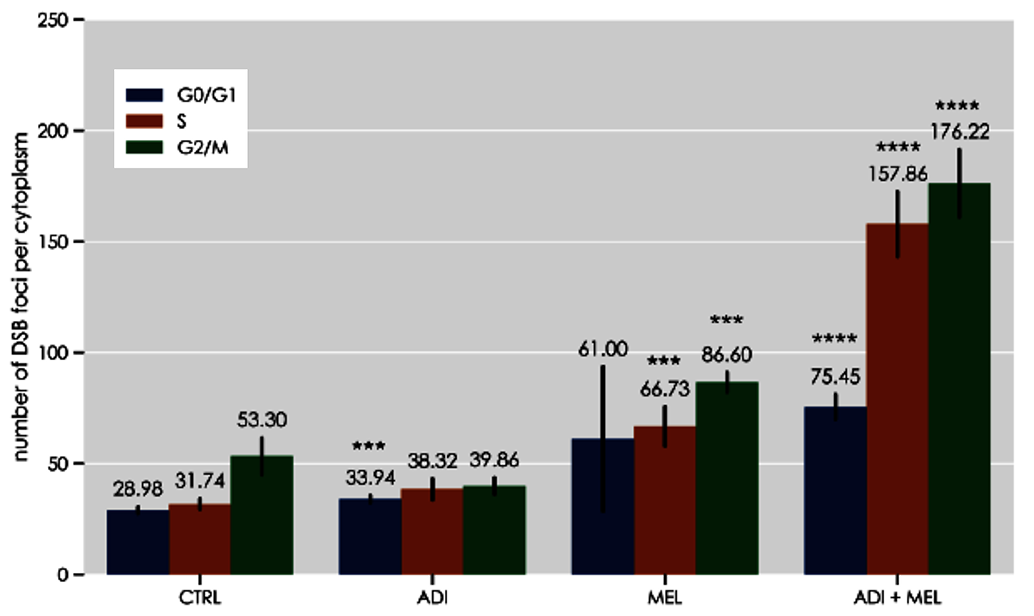


The number of cytoplasmic dSTRIDE foci per cytoplasm was quantified in 92.1 uveal melanoma cells in G0/G1, S, and G2/M phases after treatment with ADI-PEG20 (ADI), melphalan (MEL), or the combination of both agents. Untreated cells served as the control (CTRL). Bars represent mean values for each condition and cell cycle phase, with error bars indicating variation among technical replicates. **p < 0.01; ***p < 0.001; ****p < 0.0001.

**Fig. 5S: Additional validation in the ASS1 negative Mel270 cell line**

**
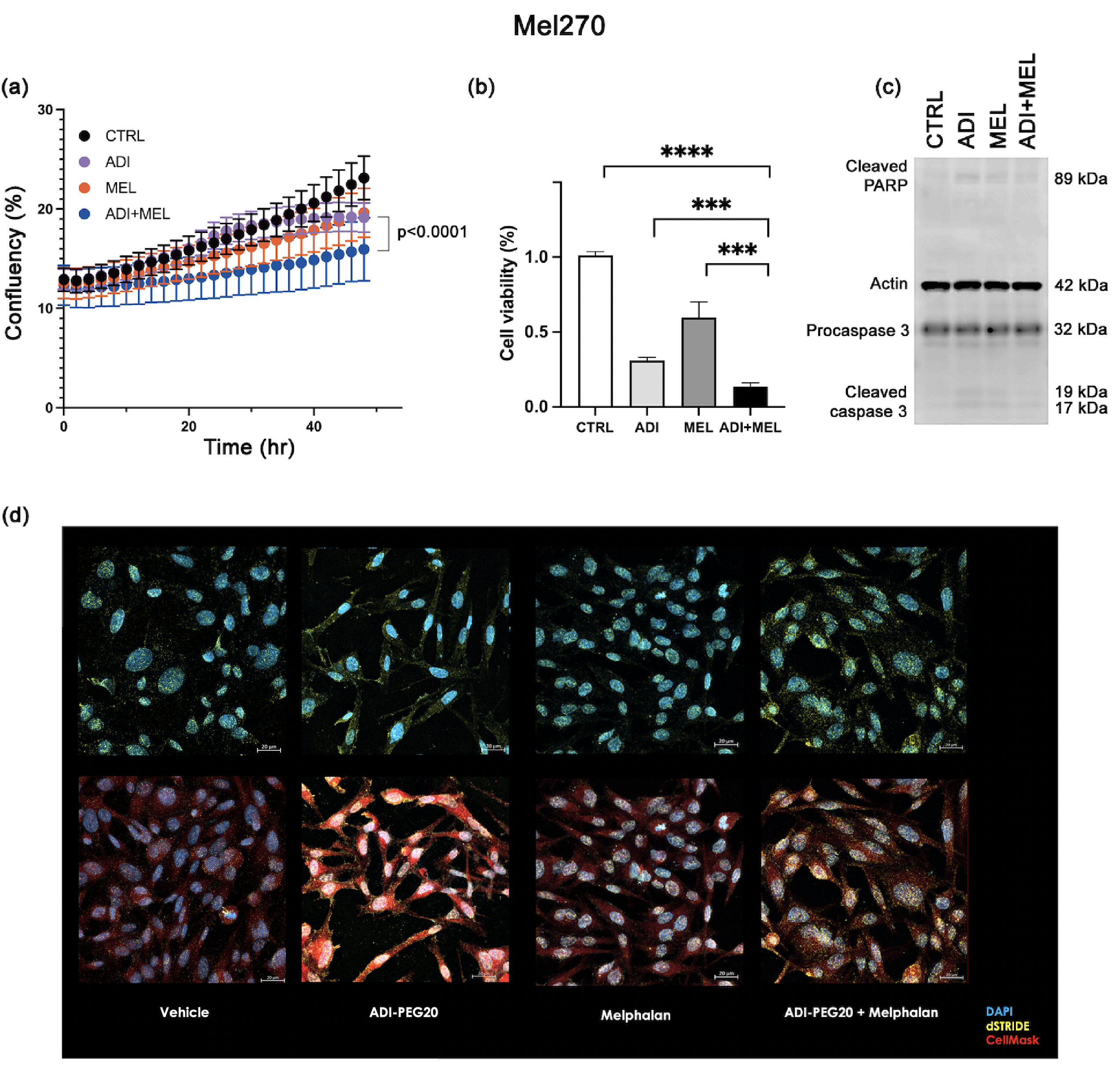
**

1. Cell proliferation in Mel270 cell line with ADI-PEG20 (750ng/ml) and melphalan (5 µM) alone or in combination and compared to untreated cells (representative of three experiments performed in triplicate). p<0.0001 for ADI vs. ADI+MEL and MEL vs ADI+MEL
2. ADI-PEG20 and melphalan increases Mel270 cell line cytotoxicity**:** control, ADI-PEG20 (750 ng/ml), melphalan (5µM), and the drug combination (n=9) with ***p=0.001, and ****=p<0.0001 (n=9)
3. Immunoblotting for PARP and caspase 3 following treatment with control, ADI-PEG20 (750 ng/ml), melphalan (5µM), and the drug combination (representative of 3 experiments)
4. ADI-PEG20 enhances melphalan-induced DNA DSBs in Mel270 cells (representative images).
